# The Evaluation of Common Bean (*Phaseolus vulgaris* L.) Genotypes under Water Stress Based on Physiological and Agronomic Parameters

**DOI:** 10.3390/plants11182432

**Published:** 2022-09-19

**Authors:** Fokion Papathanasiou, Elissavet Ninou, Ioannis Mylonas, Dimitrios Baxevanos, Foteini Papadopoulou, Ilias Avdikos, Iosif Sistanis, Avraam Koskosidis, Dimitrios N. Vlachostergios, Stefanos Stefanou, Evangelia Tigka, Anastasia Kargiotidou

**Affiliations:** 1 Department of Agriculture, School of Agricultural Sciences, University of Western Macedonia, 53100 Florina, Greece; 2 Department of Agriculture, International Hellenic University, Sindos, 57400 Thessaloniki, Greece; 3 Hellenic Agricultural Organization-Demeter, Institute of Plant Breeding and Plant Genetic Resources, Thermi, 57001 Thessaloniki, Greece; 4 Hellenic Agricultural Organization-Demeter, Industrial and Forage Crops Institute, 41335 Larissa, Greece; 5 Laboratory of Genetics and Plant Breeding, School of Agricultural Science, University of Thessaly, 38446 Volos, Greece

**Keywords:** adaptability, drought, G × E interaction, landraces, photosynthesis, yield

## Abstract

Drought affects common bean productivity, and the severity of its impact is expected to increase due to climate change. The use of versatile genotypes could contribute to securing future bean production. This study investigates the adaptability of 10 common bean genotypes of indeterminate growth type under water scarcity conditions by measuring agronomic and physiological parameters. The evaluation occurs under irrigation treatments applied at two different phenological stages (anthesis (WDA) and seed filling initiation (WDSF)). The recorded adaptabilities of the genotypes (G) showed that G10 produced the highest overall seed yield in the normal irrigation (NI) (197.22 g plant^−1^) and WDA (192.78 g plant^−1^), while the G6 had the highest yield at WDSF (196.71 g plant^−1^). For the genotype’s average mean, chlorophyll content decreased by 10.5% under drought at WDSF. Net photosynthetic rate (*P*_n_), stomatal conductance (*g*_s_), and transpiration rate (E) were reduced at WDA by 53%, 80.8%, and 61.4% and at WDSF by 43.75%, 57.7%, and 36%, respectively, while relative water content (RWC) reduced by 16.48%, on average, for both stages. G10 and G6 showed adaptability when water scarcity occurred at an early (WDA) or later stage (WDSF), respectively, providing insights into using germplasm resources to cope with the drought effect.

## 1. Introduction

Common bean (*Phaseolus vulgaris* L.) is one of the most important pulses worldwide due to its high protein content, fiber, and other essential minerals for humans [1]. Bean germplasm has significant variability, and still, extensive study of its characterization is required to reveal its breeding potential [2]. This diversity is expressed through characteristics related to plant physiology and architecture, seed traits, and yield potential [3,4] and explains why its cultivation covers a broad range of cropping systems and environments [5]. 

The growth habit of *Phaseolus vulgaris* has a wide variation [6] where the main difference in its characterization is the apical growth. This either terminates in an inflorescence (determinate types) or provides the potential for continuous indefinitely vegetative growth and flowering (indeterminate types) [7]. This trait of continuous growth may be connected with better adaptability to different environments [6], drought, or other adverse conditions due to the better sink regulation of the growing seeds [8]. Furthermore, significant genetic variability has been recorded for water use efficiency [9], which is connected with high yield under water scarcity conditions. Ceccarelli [10] concluded that high yields under stressful conditions are associated with morphological and physiological characteristics that are different from those associated with high yields under optimal conditions. Thus, the knowledge of plant responses to drought stress has been considered important for selecting genotypes tolerant of continuously changing environments [11]. In addition, genotype–environment interactions are crucial for new cultivars’ development because their stability ensures good performance across different environments [11].

Several studies indicated the severe effects of drought stress on common bean plant growth, seed yield, quality, and physiological and biochemical processes [12]. Farooq et al. [13] reported that drought limits the productivity of grain legumes at all growth stages. Its occurrence during reproductive and grain development stages is critical and usually results in significant losses in grain yield. During flowering, beans are particularly susceptible, where drought could cause important flower and pod abortion [1] and cause yield loss of over 60% [14]. Exposure to drought affects leaf area index, dry matter production, number of pods plant^−1^, number of seeds plant^−1^, hundred-seed weight, and seed yield [15,16,17,18]. Drought stress also affects plant physiological responses, resulting in a reduction in photosynthesis and transpiration rate, and intercellular carbon dioxide concentration and activates stomatal closure by the accumulation of abscisic acid (ABA), causing growth inhibition and reduced plant productivity [14,16,19,20,21,22]. The chlorophyll content is also reduced since the degradation of chlorophyll is accelerated by drought conditions and is directly related to biomass accumulation [23,24]. These responses vary depending on the stress’s frequency, duration, intensity, plant genotype, and growth stage at stress incidence [24,25].

In Greece, beans are the most important pulses, providing the backbone of the traditional Mediterranean diet, where the cultivated area has increased in recent years [26]. They are cultivated during spring and summer for their dry seed, and the most prominent area is in northern Greece, specifically at relatively high altitudes and cool temperatures. Supplemental irrigation is applied to ensure high crop yields. However, water scarcity in the Mediterranean region is a significant issue for many crops, including dry beans, as higher temperatures and more frequent drought events are projected to occur due to climate change [27,28,29,30]. 

This study used 10 common bean genotypes (including landraces and improved lines) of indeterminate growth habit, characterized by high seed quality and cultivated in important common bean production areas of northern Greece. These genotypes were tested under water-deficit conditions that occur at two different phenological growth stages to identify morphological and physiological adaptive responses contributing to sustaining yield productivity. To the best of our knowledge, there is limited or no information about the simultaneous evaluation of a number of the most important common bean genotypes of indeterminate growth cultivated in northern Greece under these adverse conditions connected to different climate change scenarios. This work aims to identify (a) the most adaptable genotypes in different cases of drought stress events that could be used directly in cultivation or as starting material in a breeding project, (b) promising genetic material with good productivity in normal and dry conditions, and/or (c) effective selection criteria that could be used in future breeding programs.

## 2. Results

### 2.1. Yield and Yield Components

The analysis of variance showed that year (Y), irrigation level (I), and genotype (G) affected seed yield (SY), number of pods plant^−1^ (NPP), and 100-seed weight (100SW), whereas only genotypes (G) differentiated within number seeds pod^−1^ (NSP) (Table 1). Regarding the two-way interactions, the Y × I was not significant, whereas the G × Y was significant for all yield components. Significant G × I was recorded for SY, NPP, and NSP. The three-way interaction was significant for yield and yield components. 

In terms of the per cent contribution to treatment, the sum of squares (%SS), Y was the main contributor in SY variation (48.8%) followed by G × I (22.3%), while G accounted for less (5.8%, Table 1). However, the SSG × I/SSG × Y ratio was 6.1-fold, and for this reason, the comparisons based on G × I interactions and the GGE biplot with a polygonal view could be useful for studying the pertinent interaction patterns. The NPP, NSP, and 100SW were moderately to highly controlled by G (26.5, 46.0, and 60.7%, respectively) and because there are secondary traits, in comparison to yield, would be assessed by their means. The 100SW, an important quality trait, was slightly affected by interaction effects (3.3–3.9%). 

Over the I and Y, the normal irrigation, N (183.06 g plant^−1^) had higher SY, whereas water-deficit treatments (water stress during anthesis, WDA; water deficit at seed filling stage, WDSF) did not differentiate between them (160.91 and 155.8 g plant^−1^, respectively; Table 2) for this parameter. The WDA treatment reduced SY by 12.1%, while the WDSF reduced SY by 14.8% (Table 2). Finally, the NPP was also significantly higher in the N in comparison to WDA and WDSF (91.54, 83.73, and 80.65 pods plant^−1^, respectively) and significantly higher for 100SW (69.72 vs. 66.45 and 66.54 g, respectively). It is also recorded that the different irrigation treatments did not affect the NSP.

**Figure 1:**
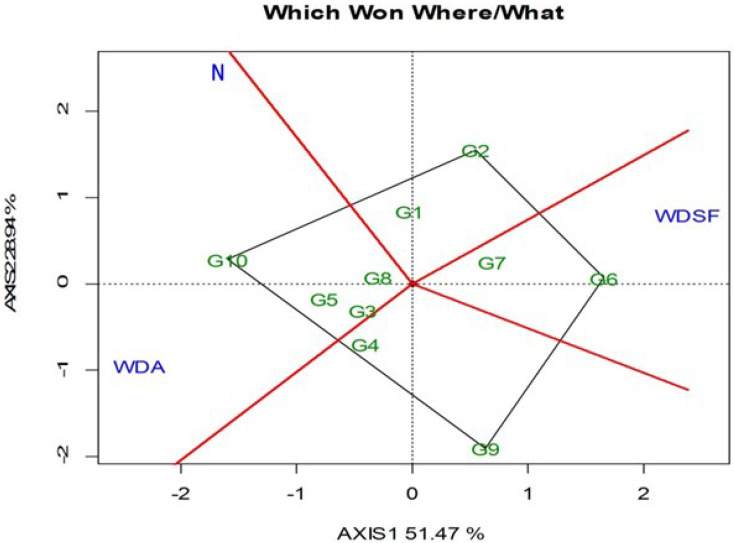
GGE biplot with the “which-won-where” pattern based on the bean seed yield across and within the three irrigation levels (N, normal irrigation; WDA, water deficit at anthesis; WDSF, water deficit at seed filling stage). The vertex genotype for each group is the one that gave the highest seed yield across the two years (2014–2015) for the irrigation levels that fall within that sector. PC1 = 51.47, PC2 = 28.94, Sum = 80.41.

The GGE biplot is based on the mean SY of each genotype for the three irrigation levels, with the “which-won-where” indicating two groupings (Figure 1). Genotype G10 followed by G5, G3, and G8 were the best performing in the normal irrigation (N) and WDA, whereas the G6 followed by G7 was the best at WDSF. The G10 was the best performing in overall SY in the N (197.22 g plant^−1^) and WDA (192.78 g plant^−1^), followed by G8 which was not significantly inferior (Table 2). The G2 and G6 was the highest yielding at WDSF (197.43 and 196.71 g plant^−1^).

**Figure 2:**
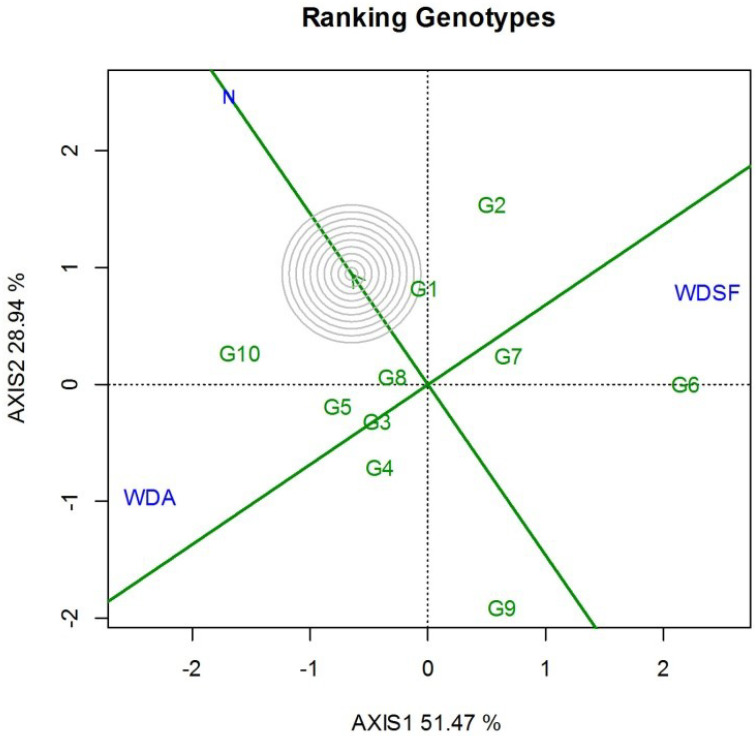
GGE biplot for ranking the ten bean genotypes with the “ideal” for seed yield and stability, based on the three irrigation levels (N, normal irrigation; WDA, water deficit at anthesis; WDSF, water deficit at seed filling stage) for two years (2014–2015). The center of the concentric circles represents the position of an ideal genotype. An ideal has both high mean seed yield and high stability. PC1 = 51.47, PC2 = 28.94, Sum = 80.41.

The GGE biplot ranking against the ideal entry for SY and stability indicated that G1 was the closest to ideal followed by G10, G8, G5, and G2, which were above the environmental mean performance as indicated by the perpendicular line to the horizontal environmental axis × (Figure 2). Genotype G6 was productive only at WDSF treatment. It was also interesting that G1 was superior in 100SW at each irrigation level (Table 2). Regarding NPP, the highest recorded values in the N were G2 and G3, whereas at WDA it was G8 and at WDSF it was G2. For NSP, the highest in the N and WDA was G10, whereas at WDSF, G8 had the highest value (Table 2). The correlation for SY ranking of the 10 genotypes among the three irrigation levels was medium and insignificant (WDA vs. WDSF, r = −0.41, *p* = ns), whereas N vs. WDA, r = 0.18, *p* = ns and N vs. WDSF, r = −0.19, *p* = ns (data not shown). In this study, G10 had the highest SY and NSP in the N and WDA and G2 and G6 the highest SY in WDSF.

### 2.2. Photosynthetic and Leaf Water Content Parameters

**Figure 3:**
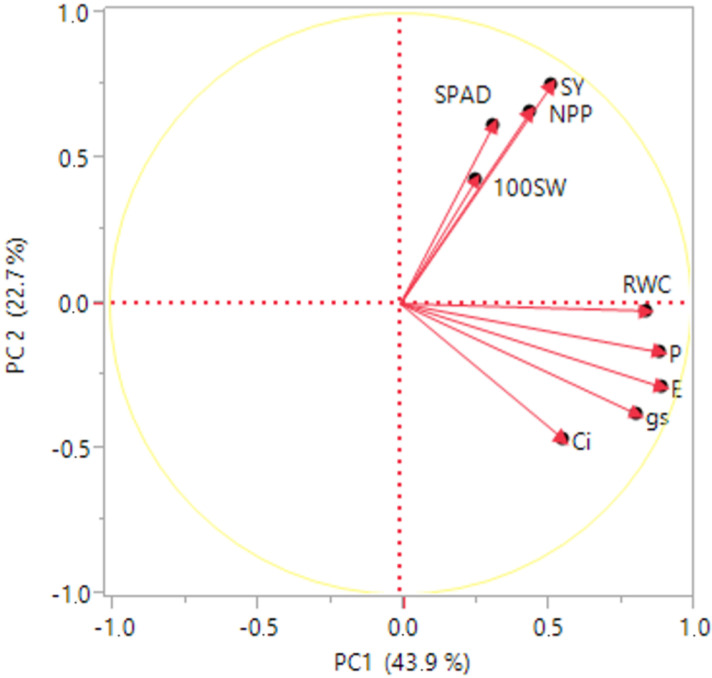
Principal component analysis (PCA) for SY, seed yield (g plant^−1^); NPP, number of pods plant^−1^; NSP, number of seeds pod^−1^; 100SW, 100 seed weight; RWC%, relative water content; SPAD, chlorophyll content; *P*_n_, net photosynthetic rate; *g*_s_, stomatal conductance; *C*_i_, intercellular CO_2_ concentration; E, transpiration rate.

Analysis of variance indicated that SPAD was affected both by irrigation treatments and genotype, but no interactions were recorded (Table 1). The different genotypes (G) and irrigation regimes (I) affected *P*_n_, *C*_i_, and RWC, while *g*_s_ and E were affected only by the different irrigation treatments. It was observed that the studied parameters were influenced mainly by I (60.9–89.2%), whereas the G effect explained only a small percentage of the variation (1–5.6%) recorded. SPAD values were reduced only at WDSF treatment compared with the N and WDA (Table 3). The *P*_n_, *g*_s_, E, and RWC were negatively affected by water scarcity at WDA and WDSF in comparison with the N. *P*_n_, g_s_,, and E reduced at WDA by 53%, 80.8%, and 61.4% and at WDSF by 43.75%, 57.7%, and 36%, respectively, while RWC reduced by 16.48%, on average, for both stages. It was also observed that *P*_n_, *g*_s_, and E were higher at WDSF in comparison with WDA, while RWC was reduced at WDA and was not differentiated from WDSF. Finally, the *C*_i_ was reduced only at WDA. The irrigation regimes (I) and the different genotypes (G) affected SPAD values. The genotypes G1 and G8 were among the best performing in the N and WDA, whereas G6 was not among the best performing at WDSF. G6 had the best net photosynthetic rate (*P*_n_) in the N followed by G10, whereas G8 at WDA and WDSF did not differentiate. For *C*_i_, g_s_, and E, most genotypes had similar values in each irrigation treatment, indicating differentiation only between treatments but _not_ within each treatment. Regarding RWC, similarly, there was no differentiation within each treatment (Table 3, Figure 3). In this study, G6 and G8 had the highest *P*_n_ in the N, while although *P*_n_ reduced under water stress, G8 and G1 sustained the net assimilation rate (*P*_n_) under water stress conditions in WDA and WDSF, respectively in comparison with the other genotypes (Table 3).

### 2.3. Associations of Yield Components with Photosynthetic Parameters and Leaf Water Status

There was a significant positive correlation between SY and NPP at WDSF (0.86, *p* ≤ 0.01) and a significant negative correlation between NPP and 100SW at WDA treatment (−0.63, *p* ≤ 0.05, Table 4). All the other seed yield components showed no relationships in either the N or water stress treatments.

The physiological parameters SPAD, *P*_n_, and E were significantly positively correlated with SY and NPP at WDSF Table 4). The RWC was positively correlated with SY and NPP at WDA (0.78, *p* ≤ 0.01 and 0.61, *p* ≤ 0.05, respectively). Furthermore, Table 4 shows the negative relationship between 100SW with *P*_n_ and RWC (−0.72, *p* ≤ 0.05 and −0.82, *p* ≤ 0.01, respectively) only under the normal irrigation treatment.

## 3. Discussion

Water stress is a major constraint on common bean productivity, plant growth, and development [31]. Greater understanding of the productive traits connected with drought tolerance is required for improving common bean susceptibility under water-limiting conditions [32]. This study investigates the connections between the indeterminate growth habit, the different bean genotypes, and the different sensitivity of the phenological stages that may occur because of a water scarcity event. Anthesis and seed filling are critical phases of the reproductive stage, and possible water-deficit events during this period cause declines in the number of seeds and pods set and eventually result in a reduction in final seed yield [33,34]. Water stress during flowering can reduce yield due to flower senescence and abortion [16], while during grain filling, the result will be the pod abortion of fertilized ovules, fewer pods and seeds per pod set [33,34], and lower average weight seed^−1^ [35]. The timing of the drought stress in relation to the bean plant growth stage affects yield, yield components, and physiological parameters [16].

Seed yield is the most important end-product of the common bean, reflecting the impact of water scarcity on grain development and connected agronomic traits [36] and most affected under these adverse conditions [37]. The decline in common bean grain production under drought conditions has been reported in several studies [18,33,34,38,39,40,41]. In this study, the SY was reduced at both WDA and WDSF, but the severity of this decline varied within the indeterminate genotypes (type IV) studied and the phenological stages that coincided with the water scarcity. Genotypes G10 and G8 had good productivity and were able to cope with water-deficit stress at anthesis (WDA), while G6 and G2 had high SY when drought stress conditions were applied at a later plant development stage, seed filling (WDSF), showing better adaptability to environments where water scarcity occurred at a later stage. The variability of SY within genotypes under limited water availability was also reported by Munoz-Perea et al. [25], who recorded a reduction in seed yield of common bean genotypes of growth habit type II and III ranging from 34 to 76%. High yield potential in the common bean could be an indirect selection criterion for drought tolerance [42]. The SY results in this study also imply, as also suggested by the GGE, the excellent adaptability of G10 and G6 when water stress conditions occur at anthesis (WDA) and seed filling (WDSF), respectively. 

Furthermore, the number of pods and seeds plant^−1^ and the 100-seed weight are generally good indicators of overall drought stress [43]. Water limitation in beans may also cause a reduction in the number of pods plant^−1^ [23,44], the number of seeds plant^−1^ [45], or both pods and seeds plant^−1^ as reported in five bush bean cultivars [46]. However, the above-mentioned traits’ sensitivity may vary; some traits such as seeds pod^−1^ are less sensitive to drought stress effects, compared with seed yield, 100-seed weight, pods plant^−1^, and leaf chlorophyll content [47]. Our findings showed that drought reduced NPP and 100SW at both phenological stages where water deficit occurred (anthesis and seed filling). Interestingly, the irrigation treatments did not affect the number of seeds developed per pod (NSP) irrespective of the growth stage applied; this parameter differentiated only between the studied genotypes as a characteristic of their genotype. Similarly, Darkwa et al. [43] reported a significant decline in the number of pods plant^−1^, 100-seed weight, and seed yield under drought stress in common beans, while seed number per pod proved less sensitive to drought stress and was not affected. This may be an indication of plant effort for sink strength maintenance; Hageman and Volenburgh [8] outline that wild common beans with indeterminate growth habit enhance growth and seed filling under drought and sustain their ability to produce another flush of flowers when the conditions are improved. 

Growth habit plays a role in coping with water scarcity, and it seems that the indeterminate growth type is an important trait for adaptation under these conditions [42]. The landraces and improved lines studied had indeterminate growth habit IV, resulting in continuous blooming and producing more biomass than the other types of common bean [48]. Sink strength maintenance may contribute to better yield performance in common beans [8]. The indeterminate growth habit of the studied genotypes can continuously produce under optimal conditions, which probably provide flexibility to cope with drought stress. Rosales-Serna et al. [42] reported a better adaptation of the indeterminate cultivars under water-limiting conditions than the determinate cultivars.

Concerning physiological responses, it is known that water deficit triggers senescence symptoms connected with chlorophyll degradation and the reduction of chlorophyll content in the leaves of stressed plants [32]. Drought affected chlorophyll content in common beans [16]. As a result, non-stressed plants had higher chlorophyll than stressed ones, and genotypes with higher chlorophyll content may produce higher yields [43]. Rosales-Serna et al. [42] established that chlorophyll content, in two contrasting growth types (I and III) of common beans, expressed as SPAD readings, could discriminate the different cultivars; however, its use in selection for drought resistance may be limited. Similarly, our findings showed that chlorophyll content was preserved at anthesis (WDA), and the degradation started at seed filling, since the decline was observed only at the WDSF stage, expressed as SPAD readings. Chlorophyll content differentiated between the different genotypes, but it was not affected by irrigation treatments, and only at WDSF was chlorophyll content correlated with SY. 

Regarding the net assimilation rate, Rosales et al. [36] reported that the early response and fine tuning of stomatal conductance, CO_2_ diffusion, and fixation maintained seed productivity under drought conditions. Mathobo et al. [16] mentioned that drought stress in beans resulted in a reduction in stomatal conductance (g_s_). This is due to stomatal closure, which prevents CO_2_ from entering the leaf photosynthetic carbon assimilation and is decreased in favor of photorespiration. Our findings showed that the drought stress at anthesis reduced *P*_n_ in all studied genotypes; water limitation at anthesis (WDA) caused immediate stomatal closure (*g*_s_) to control evapotranspiration losses (E) due to water stress. The decline in photosynthetic activity observed at anthesis (WDA) was higher in comparison with the water stress applied at a later developmental stage (WDSF). Despite this general decline a genetic variability observed where G8 and G1 sustain net assimilation rate (*P*_n_) in WDA and WDSF, respectively in comparison with the other genotypes

Regarding physiological indices and their correlations with yield components, SPAD, *P*_n_, and E correlated with SY only at WDSF, whereas only RWC correlated with SY at WDA. This could be an indication that although the source can affect sink development (growing seeds), there are cases where sink growth is not directly or exclusively linked to source strength [8]. The NPP was also related to WDSF, similarly to SY. The 100SW was negatively related to SY in the N, and there was no relationship with indices within other treatments. The physiological indices succeeded in indicating differences among the genotypes only at WDSF. Thus, the specifically adapted G6 to WDSF was among the best performing in *P*_n_, *C*_i_, *g*_s_, and E. Genotype G10 showed good performance under sufficient water supply (N), and water deficit occurred at anthesis (WDA), indicating a genetic background that can successfully manage sink strength by forming fewer pods (NPP) but more seeds per pod (NSP). This genotypic response may provide better adaptation when drought stress occurs early, during anthesis. The recovery of the water availability at a post-anthesis stage seems unable to maintain productivity. Genotype G1 had the lowest NSP at WDA and WDSF, but the largest seed size showed a likely ability to sustain sink strength across different environments. Finally, the highest RWC values were obtained in the N as a useful indicator showing the state of the water balance of a plant and affected by drought stress; this caused an immediate decline in RWC regardless of the phenological stage. The RWC decreased with the severity of drought stress as a mechanism to sustain water balance under drought conditions [49].

## 4. Materials and Methods

### 4.1. Genetic Material

Seven landraces from different geographical origins, characterized by stable yields, and three improved lines of bean *Phaseolus vulgaris* L. (Table 5) were used. Each landrace, cultivated continuously for over 30 years by each farmer avoiding seed mixing, was initially collected (500 g sample) from farmers’ stocks. The improved lines originated from the intragenotypic selection under honeycomb design at a low density from two of the seven landraces studied (Table 5) as described in Tokatlidis et al. [50] and Tokatlidis and Vlachostergios [51]. All landraces and improved lines belonged to the white-large-seed type, with a hundred-seed weight (100SW) over 60 g and indeterminate climbing type IV.

### 4.2. Site and Experiment Setup

**Figure 4:**
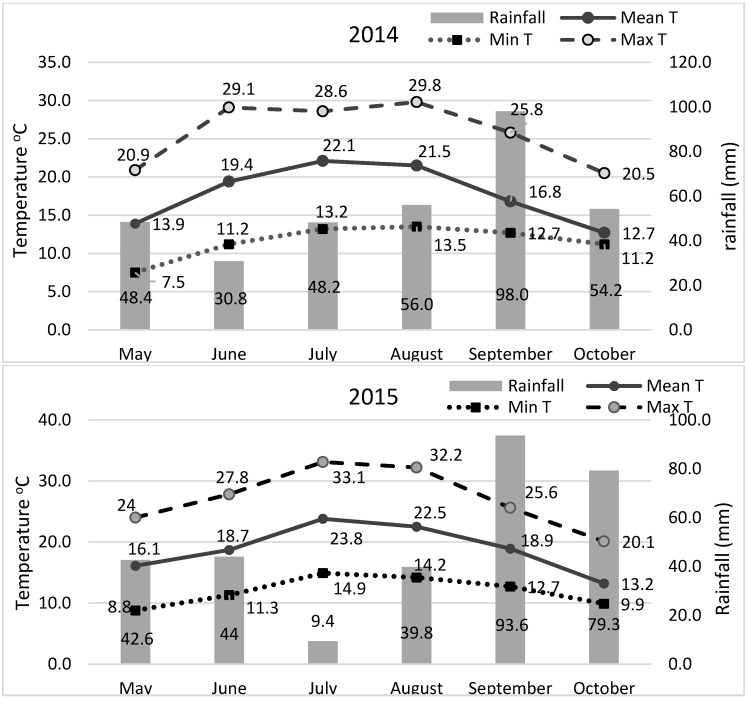
Climatic conditions during the two successive cultivation periods during the years 2014 and 2015.

The experiments were conducted during two successive crop seasons in 2014 and 2015 (sowing time early May; harvest late October) at the experimental farm of the Department of Agriculture of the University of Western Macedonia, Florina, Greece (686 m a.s.l.; 21°23′ E, 40°46′ N) Climatic conditions during the two cultivation periods are shown in Figure 4. Weather conditions were quite different between the two growing seasons. During 2014, precipitation was more uniform across the growing season, whereas 2015 was drier during July–August and warmer throughout the season (Figure 4). The average monthly precipitation was higher in July (48.2 mm) and August (56.0 mm) in 2014 compared to 2015 (9.4 mm and 39.8 mm, respectively). The recorded temperature in July and August was lower in 2014 (22.1 °C and 21.5 °C, respectively) compared to 2015 (23.8 °C and 22.5 °C, respectively). The soil was sandy loam with pH 6.67, organic matter 17.6 g kg^–1^, N-NO_3_ 80.0 mg kg^–1^, Olsen P 82.3 mg kg^–1^, and K 214 mg kg^–1^.

### 4.3. Experimental Design and Crop Management

**Figure 5:**
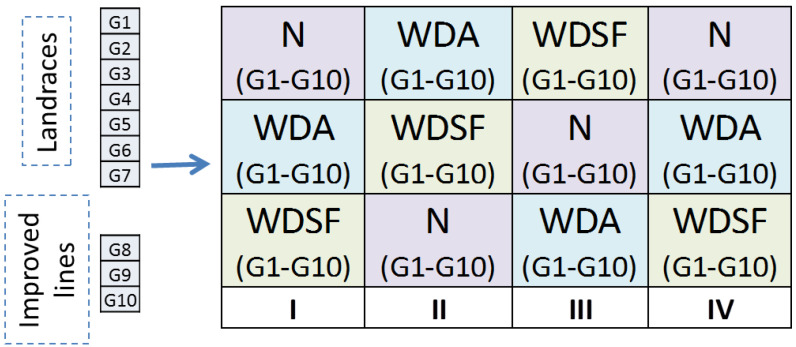
The split-plot design with 4 replications, the main plot with the irrigation treatments (N, WDA and WDSF) and the sub-plots with the 10 genotypes randomized within the main plots.

The field experiments were arranged according to a split-plot design with four replications: the main plots were the three irrigation levels and the subplots of the ten genotypes (Figure 5). Each subplot was 3 m long, with 4 rows 0.80 m apart and 0.60 m plant-to-plant distance. Due to all the genotypes’ climbing growth habit, each plant was supported with a common cane supplemented with an iron pole. Water supply was imposed with a drip-irrigation system, with the drippers spaced at 60 cm intervals, along the lines, and water supply of 4 L h^−1^, starting at 55 days after planting (DAP) and altered according to each treatment as follows: (a) normal (N), where the plants were watered according to optimum requirement every 4 days for 2 h until physiological maturity (BBCH 75); (b) water deficit during anthesis (WDA), where irrigation was suspended for 12 days just after the beginning of anthesis (BBCH 60) and afterwards watered similarly to the N; and (c) water deficit during seed filling initiation stage (WDSF), where irrigation was suspended for 12 days at the beginning of the seed filling period (BBCH 70) and afterwards watered similarly to the N. 

The experiments were fertilized at a rate of 60–40–40 kg ha^−1^ of N-P-K applied to the soil before sowing and with N top dressing of 60 k ha^−1^ at the proper plant growth stage in both years. Weeds were controlled using mechanical cultivation complemented with hand weeding. Pests were controlled using conventional chemical means (deltamethrin 2.5%, 4th leaf). Seeds were sown in both years approximately on May 1 and harvested in mid-October at maturity time when pods had lost their pigmentation and started the drying process (BBCH 85–89). 

### 4.4. Agronomic Measurements (Yield and Yield Components)

Crop sampling occurred at maturity in mid-October, as mentioned above. In every plot, border plants were omitted in harvest and all observations and measurements were taken from the central rows of each subplot. In total, eight plants were harvested per subplot. Traits recorded were seed yield (SY, g plant^−1^), determined at 10% moisture content, number of pods plant^−1^ (NPP), number of seeds pod^−1^ (NSP), and 100-seed weight (100SW, g).

### 4.5. Leaf Gas Exchange and Chlorophyll Content Measurements

Net photosynthetic rate, (*P*_n_*)*, stomatal conductance (*g*_s_), transpiration rate (E), and intercellular CO_2_ concentration (*C*_i_) were evaluated using a portable open gas exchange system (LI-400 XT, Li-Cor, Lincoln, NE, USA). A light intensity of 1500 μmol m^−2^ s^−1^ (provided by 6400-02B led light source), block temperature of 25 °C, and 400 ppm CO_2_ were fixed for all measurements in both years. The measurements were taken in the middle leaflet of the fifth fully expanded trifoliate leaf from the top of the plants. In each subplot, four plants were measured at the onset of each water-deficit treatment (well-hydrated plants, day 0), and on the 12th day of each water deficit treatment (day 12), between 09:00 and 13:00 to avoid high vapor pressure deficit and photoinhibition at midday. At the same measurement period, in the same plants for each subplot and the same fully expanded trifoliated leaf, chlorophyll content was determined with a SPAD-502 chlorophyll meter (Minolta, Japan) in the central leaflet. Each SPAD value obtained was the average of 6 readings (3 on each side of leaf midrib). The SPAD-502 m was calibrated using the reading checker supplied by the manufacturer.

### 4.6. Leaf Water Content Determination

After measuring photosynthetic parameters at the end of each water-deficit treatment (day 12), the same trifoliate leaves from the same four plants per subplot were used to evaluate RWC. The side leaflet from each leaf was excised between 11:00 and 12:00, covered with aluminum foil, and stored in a portable freezer, avoiding direct contact with ice. A sharp cork borer was used in the laboratory to cut 8 leaf discs 12 mm in diameter, avoiding the mid-rib and major veins, and weighed (fresh weight, FW). The leaf discs were floated on distilled water for more than 8 h in darkness at 4 °C, then wiped off with absorbent paper and weighed (turgid weight, collect TW). Leaf disc samples were dried in an oven at 70 °C for 48 h to determine the dry weight (DW). RWC was estimated according to the equation RWC (%) = [(FW-DW)/(TW-DW)] × 100.

### 4.7. Statistical Analysis

Data were subjected to over-year analysis of variance (ANOVA) as a quadruplicated split-plot design with irrigation levels (I) as the main plot factor and genotypes (G) as sub-plot. The Shapiro–Wilk test for normality showed that the variables were normally distributed, whereas the ANOVA’s assumptions for the equality of the error variances and residual normality were met by Levene’s test and the normal quantile–quantile (QQ) plot method, respectively [52]. The differences between mean values were evaluated by using LSD (Fischer’s least significance test) [53] at α = 0.05. The analyses were performed using the statistical software IBM SPSS package v. 23 (IBM Corp., New York, NY, USA). Principal component analysis (PCA) for traits was determined. The first two principal components, PC1 and PC2, were derived from the eigenvalue decomposition of the correlation matrix of the variables. Based on the eigenvector values, those explaining most of the variation were used to print the respective scatterplots.

Genotype plus genotype × environment (GGE) biplot analysis was used for analyzing G × Y interactions and ranking cultivars for yield and stability [54]. The advantage of the GGE biplot model is removing the noise caused by the environment’s main effect and generating biplots based on G + GE, which are relevant to cultivar evaluation [55,56]. The free software package performed the GGE biplot analysis, GGE Biplots in R version 1.0–8 [57]. 

## 5. Conclusions

The evaluation revealed two genotypes with high versatility under drought conditions that could contribute to common bean adaptability in the upcoming climate change scenario and could be used directly in cultivation or as starting material in a breeding project. Specifically, G10 showed good productivity when water deficit occurs at an early stage of reproductive development (WDA, anthesis), while G6 showed adaptability when drought events happen at a later stage (WDSF, seed filling). This is probably connected with the ability of each genotype to maintain sink strength and the balance of source availability under water-limiting conditions. Further study of genotype × environment interactions is important for revealing genotypes showing good performance and stability across conditions with adverse conditions and provide useful information for breeders and agronomists. 

## Figures and Tables

**Table 1:** Mean squares analysis of variance (ANOVA) and percentage (%) contribution regarding the treatment sum of squares (%SS) of the ten bean genotypes as affected by three irrigation levels over the two years (2014–2015).

Source	df	SY ^1^	NPP	NSP	100SW	SPAD	*P*_n_	*g*_s_	*C*_i_	E	RWC %
Year (Y)	1	205,427.8 **	17,802.0 **	0.12 ^ns^	5393.2 **	2109.3 **	0.00 ^ns^	0.25 **	2527.24 *	0.00 ^ns^	133.2 **
Irrigation level (I)	2	33,460.6 **	5040.8 **	0.06 ^ns^	554.4 **	876.6 **	5476.36 **	7.24 **	125,621.59 **	223.74 **	11,524.3 **
Genotype (G)	9	24,533.8 **	22,683.7 **	15.94 **	12,793.8 **	1144.4 **	118.19 **	0.33 ^ns^	11,498.05 **	6.25 ^ns^	124.6 ^ns^
Y × I	2	1357.6 ^ns^	177.0 ^ns^	0.68 ^ns^	78.80 ^ns^	188.7 ^ns^	35.59 *	1.51 **	17,547.08 **	5.35 **	1.7 ^ns^
G × Y	9	14,960.3 **	3833.8 *	3.97 **	700.5 **	462.6 ^ns^	71.90 ^ns^	0.23 ^ns^	14,565.47 **	4.95 ^ns^	89.8 ^ns^
G × I	18	93,724.5 **	17,861.7 **	7.31 **	711.2 ^ns^	633.7 ^ns^	223.47 **	0.60 ^ns^	15,748.72 ^ns^	8.97 ^ns^	385.8 **
G × I × Y	18	47,619.9 **	18,072.5 **	6.59 **	828.5 **	858.7 ^ns^	217.25 **	0.71 ^ns^	18,857.15 *	14.64 *	662.8 **
	Percent contribution to treatment sum of squares (%SS)										
SS_Y_		48.8	20.8	0.3	25.6	33.6	0.0	2.3	1.2	0.0	1.0
SS_I_		7.9	5.9	0.2	2.6	14.0	89.2	66.6	60.9	84.8	89.2
SS_G_		5.8	26.5	46.0	60.7	18.2	1.9	3.1	5.6	2.4	1.0
SS_Y×I_		0.3	0.2	2.0	0.4	3.0	0.6	13.9	8.5	2.0	0.0
SS_G×Y_		3.6	4.5	11.5	3.3	7.4	1.2	2.1	7.1	1.9	0.7
SS_G×I_		22.3	20.9	21.1	3.4	10.1	3.6	5.5	7.6	3.4	3.0
SS_G×I×Y_		11.3	21.1	19.0	3.9	13.7	3.5	6.5	9.1	5.5	5.1

*, **: significance at *p* < 0.05 and 0.01 level, respectively; ^ns^: non-significant. ^1^ SY: seed yield; NPP: number of pods plant^−1^; NSP: number seeds pod^−1^; 100SW: 100-seed weight; *P*_n_: net assimilation rate; *g*_s_: stomatal conductance; *C*_i_: intracellular CO_2_; E: transpiration rate; RWC: relative water content.

**Table 2:** Comparisons for seed yield and yield components of the ten bean genotypes as affected by three irrigation levels (N, normal irrigation, WDA, water stress during anthesis; WDSF, water stress during seed filling stage) for two years (2014–2015).

Irrigation Level	Genotype	SY-Overall		SY-2014 ^2^		SY-2015		NPP		NSP		100SW	
		(g plant^−1^)						(Pods plant^−1^)		(Seeds pod^−1^)		(g)	
**N**	G1	191.96	ab ^1^	193.37	e–h	190.56	a	90.13	d–k	2.62	j–l	82.98	a
	G2	195.68	a	230.69	a–d	160.68	bc	107.88	ab	2.64	i–l	69.13	e–h
	G3	182.58	a–d	212.18	b–f	152.99	b–e	105.25	a–c	2.62	j–l	66.87	f–i
	G4	178.47	a–f	200.73	c–h	156.22	b–e	86.00	e–l	2.96	d–j	70.92	d–g
	G5	186.07	a–c	232.16	a–d	139.98	b–h	99.13	b–e	2.87	e–j	64.25	h–k
	G6	172.32	b–g	197.58	d–h	147.05	b–g	98.63	b–f	2.82	f–k	61.86	i–l
	G7	180.81	a–e	218.27	b–f	143.36	b–g	87.13	e–l	2.95	d–j	70.87	d–g
	G8	186.49	a–c	223.19	a–e	149.79	b–f	98.00	b–g	3.30	b–d	57.83	l
	G9	159.03	e–i	187.05	e–i	131.00	e–h	71.38	m–p	2.78	g–l	79.56	ab
	G10	197.22	a	235.08	a–c	159.36	bc	71.88	m–p	3.81	a	72.90	c–e
	**Mean**	**183.06**	**A**	**213.03**		**153.10**		**91.54**	**A**	**2.94**	**A**	**69.72**	A
**WDA**	G1	153.95	g–i	167.81	hi	140.08	b–h	76.63	k–o	2.50	kl	80.66	ab
	G2	146.89	h–j	191.70	e–h	102.08	i–k	84.63	g–m	2.78	g–l	61.27	jkl
	G3	161.99	d–i	200.06	c–h	123.93	g–j	82.88	h–n	3.17	c–f	60.46	kl
	G4	168.98	c–h	201.32	c–h	136.64	c–h	86.13	e–l	2.98	c–i	66.76	f–i
	G5	158.30	f–i	199.16	c–h	117.43	h–k	91.00	d–j	2.78	g–l	64.57	h–k
	G6	127.14	jk	129.57	jk	124.72	f–j	81.88	i–n	2.96	d–j	53.74	m
	G7	139.67	i–k	182.05	f–i	97.30	k	70.38	n–q	2.72	g–l	71.79	c–f
	G8	186.68	a–c	216.74	b–f	156.63	b–d	103.88	a–d	3.07	c–g	58.81	lm
	G9	172.72	b–g	198.86	c–h	146.58	b–g	74.75	l–p	3.03	c–h	76.08	bc
	G10	192.78	ab	239.34	ab	146.22	b–g	85.13	f–m	3.20	c–e	70.39	e–g
	**Mean**	**160.91**	**B**	**192.66**		**129.20**		**83.73**	**B**	**2.92**	**A**	**66.45**	**B**
**WDSF**	G1	161.06	d–i	173.63	g–i	148.48	b–g	82.50	h–n	2.46	l	82.79	a
	G2	197.43	a	232.49	a–d	162.36	b	117.50	a	2.62	j–l	64.77	h–k
	G3	126.14	j–l	153.12	i–j	99.16	jk	78.13	j–o	2.68	h–l	60.12	kl
	G4	130.62	jk	166.58	h–j	94.67	k	67.63	o–q	2.93	e–j	66.31	g–j
	G5	103.81	l	91.03	l	116.58	h–k	62.25	pq	2.66	i–l	61.30	j–l
	G6	196.71	a	255.64	a	137.79	b–h	95.88	b–h	3.32	bc	60.95	kl
	G7	166.23	c–h	184.98	f–i	147.48	b–g	75.88	l–p	3.19	c–e	69.19	e–h
	G8	183.17	a–d	233.32	a–d	133.02	d–h	92.00	c–i	3.63	ab	57.91	lm
	G9	171.38	b–g	210.07	b–g	132.69	d–h	78.00	j–o	2.89	e–j	75.89	b–d
	G10	122.30	kl	118.54	kl	126.06	f–i	56.75	Q	3.21	c–e	66.15	g–j
	**Mean**	**155.88**	**B**	**181.90**		**129.80**		**80.65**	**B**	**2.96**	**A**	**66.54**	**B**

^1^ Different letters represent significantly different means for *p* < 0.05. ^2^ SY: seed yield; NPP: number of pods plant^−1^; NSP: number seeds pod^−1^; 100SW: 100-seed weight.

**Table 3:** Comparison of SPAD, *P*_n_, *C*i, *g*s, E, RWC parameters of ten bean genotypes as affected by three irrigation levels (N, normal irrigation conditions; WDA, water stress during anthesis; WDSF, water stress during seed filling stage) over two years (2014–2015).

Irrigation Level	Genotype	SPAD		*P*_n_^2^		*C*_i_		*g*_s_		E		RWC	
				µmol CO_2_ m^−2^s^−1^		µmol CO_2_ mol^−1^		mol H_2_O m^−2^s^−1^		mmol H_2_O m^−2^s^−1^		(%)	
**N**	G1	43.88	a–c ^1^	18.38	d	248.21	a–c	0.5	b–d	3.64	bc	85.88	e
	G2	36.24	f–i	19.67	cd	233.62	c–f	0.38	d–f	3.34	cd	87.9	c–e
	G3	39.05	b–i	21.26	a–c	253.23	a–c	0.55	a–c	3.80	bc	90.35	a–c
	G4	41.83	a–f	20.64	b–d	236.82	b–f	0.42	cd	3.24	cd	89.84	a–c
	G5	44.91	ab	19.30	cd	256.86	a–c	0.53	a–d	4.14	ab	88.75	b–d
	G6	42.84	a–d	23.03	a	253.89	a–c	0.69	a	4.58	a	91.8	a
	G7	42.74	a–d	20.59	b–d	246.53	a–c	0.49	b–d	4.20	ab	88.99	b–d
	G8	46.58	a	22.61	ab	242.89	a–d	0.61	ab	3.83	bc	90.73	ab
	G9	41.24	a–f	19.77	cd	249.02	a–c	0.46	b–d	3.87	bc	86.93	de
	G10	39.94	b–g	21.31	a–c	257.54	ab	0.54	a–d	3.72	bc	89.97	a–c
	**Mean**	**41.92**	**A**	**20.65**	**A**	**247.86**	**A**	**0.52**	**A**	**3.83**	**A**	**89.11**	**A**
**WDA**	G1	43.50	a–c	9.84	i-m	214.87	e–g	0.12	gh	1.73	g–i	74.07	f–j
	G2	40.24	b–g	10.68	f–l	167.61	I	0.10	gh	1.43	i	74.02	g–j
	G3	39.40	b–i	9.84	i–m	199.71	gh	0.10	gh	1.45	i	75.34	f–h
	G4	41.43	a–f	9.55	j–m	182.95	hi	0.10	gh	1.40	i	75.03	f–h
	G5	41.04	a–f	9.08	k–m	213.41	fg	0.10	gh	1.40	i	74.47	f–i
	G6	43.48	a–c	8.52	Lm	201.10	gh	0.09	gh	1.41	i	73.67	h–j
	G7	36.94	d–i	9.41	j–m	203.33	gh	0.10	gh	1.36	i	73.62	h–j
	G8	43.71	a–c	11.44	e–j	199.03	gh	0.13	gh	1.82	f–i	76.67	fg
	G9	43.28	a–c	10.58	g–l	200.02	gh	0.11	gh	1.60	hi	75.68	f–h
	G10	38.18	c–i	8.00	M	198.23	gh	0.08	h	1.21	i	74.76	f–h
	**Mean**	**41.12**	**A**	**9.70**	**C**	**198.03**	**C**	**0.10**	**C**	**1.48**	**C**	**74.73**	**B**
**WDSF**	G1	39.73	b–h	13.04	e	237.40	b–e	0.22	f–h	2.46	ef	74.59	f–h
	G2	39.15	b–i	12.75	e–g	249.70	a–c	0.23	e–h	2.53	e	76.83	f
	G3	33.70	i	11.62	e–j	243.86	a–d	0.19	gh	2.50	e	71.45	j
	G4	36.40	e–i	9.64	j–m	245.87	a–c	0.16	gh	2.21	e–h	71.72	ij
	G5	33.81	hi	10.15	h–m	243.87	a–d	0.16	gh	2.32	e–g	75.27	f–h
	G6	39.66	b–h	11.37	e–k	263.94	a	0.24	e–h	2.55	e	73.44	h–j
	G7	34.98	g–i	12.46	e–h	221.38	d–g	0.20	gh	2.37	e–g	73.92	g–j
	G8	42.35	a–e	12.00	e–i	253.07	a–c	0.25	e–g	2.68	de	75.85	f–h
	G9	40.68	a–g	12.99	ef	242.42	a–d	0.39	cde	2.55	e	74.59	f–h
	G10	34.81	g–i	10.22	h–m	249.94	a–c	0.15	gh	2.32	e–g	73.5	h–j
	**Mean**	**37.53**	**B**	**11.62**	**B**	**245.14**	**A**	**0.22**	**B**	**2.45**	**B**	**74.11**	**B**

^1^ Different letters represent significantly different means for *p* < 0.05. ^2^ *P*_n_: net assimilation rate; *g*_s_: stomatal conductance; *C*_i_: intracellular CO_2_; E: transpiration rate; RWC: relative water content.

**Table 4:** Correlation coefficients for SY, NPP, NSP, 100SW, RWC (%), SPAD, *P*_n_, *g*_s_, *C*_i_, E of the ten bean genotypes as affected by three irrigation levels [normal irrigation (N), water stress during anthesis (WDA) and water stress during seed filling stage (WDSF)] over two years (2014–2015). Correlations coefficients (n = 10) superscripted with * or ** were significant at *p* ≤ 0.05 or 0.01.

	SY			NPP			NSP			100SW		
	N	WDA	WDSF	N	WDA	WDSF	N	WDA	WDSF	N	WDA	WDSF
SY ^1^				0.30	0.49	0.86 **	0.32	0.57	0.31	−0.08	0.22	0.09
NPP	0.31	0.49	0.86 **				−0.52	0.37	0.00	−0.59	−0.57	−0.10
NSP	0.31	0.57	0.31	−0.52	0.37	0.00				−0.19	−0.43	−0.50
100SW	−0.08	0.21	0.09	−0.59	−0.63 *	−0.10	−0.19	−0.43	−0.50			
SPAD	−0.16	0.01	0.80 *	−0.08	0.32	0.63 *	0.18	−0.11	0.30	−0.26	−0.08	0.22
*P*_n_	−0.17	0.17	0.69 *	0.12	0.29	0.63 *	0.42	−0.09	−0.20	−0.72 *	−0.01	0.49
*C*_i_	−0.09	0.00	0.23	−0.25	−0.13	0.31	0.28	−0.21	0.30	−0.02	0.34	−0.50
*g*_s_	−0.12	0.14	0.59	0.14	0.32	0.39	0.26	−0.27	0.07	−0.55	0.12	0.35
E	−0.38	0.14	0.72 *	0.06	0.29	0.70 *	0.00	−0.24	0.28	−0.37	0.12	−0.10
RWC	−0.04	0.78 **	0.48	0.25	0.61 *	0.52	0.40	0.61	0.00	−0.82 **	−0.09	0.06

*, and **: significance at *p* < 0.05 and 0.01, respectively; ^1^ SY: seed yield; NPP: number of pods plant^−1^; NSP: number seeds pod^−1^; 100SW: 100-seed weight; *P*_n_: net assimilation rate; *g*_s_: stomatal conductance; *C*_i_: intracellular CO_2_; E: transpiration rate; RWC: relative water content.

**Table 5:** Genotype numeration and sources of bean landraces and improved lines evaluated.

Genotype No	Name	Landrace/Improved Lines	Origin	Growth Type	Seed Type
G1	Agios Germanos	Landrace	Agios Germanos-Prespes-Greece	IV	White-large
G2	Florina	Landrace	Florina-Greece	IV	White-large
G3	Nacolets	Landrace	Nacolets-North Macedonia	IV	White-large
G4	Laimos	Landrace	Laimos-Prespes-Greece	IV	White-large
G5	Xrisoupoli	Landrace	Xrisoupoli-Kavala-Greece	IV	White-large
G6	Kastoria	Landrace	Korestia Kastorias-Greece	IV	White-large
G7	Plati	Landrace	Plati Prespes-Greece	IV	White-large
G8	Improved line	Improved line	Selection from genotype 1	IV	White-large
G9	Improved line	Improved line	Selection from genotype 5	IV	White-large
G10	Improved line	Imporved line	Selection rom genotype 5	IV	White-large

## Data Availability

Not applicable.
